# Fe_1−x_S Modified TiO_2_ NPs Embedded Carbon Nanofiber Composite via Electrospinning: A Potential Electrode Material for Supercapacitors

**DOI:** 10.3390/molecules25051075

**Published:** 2020-02-27

**Authors:** Bishweshwar Pant, Hem Raj Pant, Mira Park

**Affiliations:** 1 Department of Biomedical Sciences and Institute for Medical Science, Jeonbuk National University Medical School, Chonbuk 54907, Korea; bisup@jbnu.ac.kr; 2 Department of Applied Sciences, Tribhuvan University, Kathmandu 44600, Nepal; hempant@ioe.edu.np

**Keywords:** Fe_1−x_S-TiO_2_, carbon nanofibers, composite, electrospinning, energy storage

## Abstract

Fe_1−x_S-TiO_2_ nanoparticles embedded carbon nanofibers (Fe_1−x_S-TiO_2_/CNFs) composite as a supercapacitor electrode material has been reported in the present work. The Fe_1−x_S-TiO_2_/CNFs composite was fabricated by electrospinning technique followed by carbonization under argon atmosphere and characterized by the state-of-art techniques. The electrochemical studies were carried out in a 2 M KOH electrolyte solution. The synthesized material showed a specific capacitance value of 138 F/g at the current density of 1 A/g. Further, the capacitance retention was about 83%. The obtained results indicate that the Fe_1−x_S-TiO_2_/CNFs composite can be recognized as electrode material in supercapacitor.

## 1. Introduction

Due to the ever-increasing energy demand, developing efficient and environmentally friendly energy storage devices has become one of the major tasks among the researchers. Supercapacitors have been recognized as a reliable and efficient energy storage device in recent years [1,2,3]; however, the performance of supercapacitors must be significantly improved to fulfill the future global energy demand. Since the electrochemical properties of electrode play an important role in the overall performance of supercapacitor, the fabrication of high-performance electrode material with an excellent electrochemical performance is essential [1,4].

Recently, transition metal oxides such as Co_3_O_4_, Fe_2_O_3_, RuO_2_, NiO, ZnO, TiO_2_, MgO, MnO_2_ have been applied as electrode material for supercapacitors [1,3,5,6]. Among them, TiO_2_ as one of the typical pseudocapacitance materials has attracted immense interest for supercapacitors due to its high stability, low-cost, excellent electrochemical stability, and nontoxicity [4,7,8]. However, one major issue is the poor electric conductivity that limits its power density and rate capability [9]. To overcome this problem, most of the reports have been focused on combining TiO_2_ with conductive carbon materials such as carbon nanofibers [4], graphene [7], and carbon nanotubes [10]. The carbon nanofibers offer mechanical and physicochemical stability of the composite, as well as prevent the aggregation of metal oxide nanoparticles [3,11]. In addition, the carbon nanofibers possess high electrical conductivity, high charge transfer ability, large surface area and mesoporosity, and high electrolyte accessibility [12]. Therefore, integrating TiO_2_ into the carbon structures shows the combined effect of the pseudocapacitance of metal oxide and double-layer capacitance of carbon. In our previous study [4], we fabricated TiO_2_ NPs embedded carbon nanofibers via the electrospinning technique and applied as a supercapacitor electrode. It was observed that after incorporating TiO_2_ into carbon fibers, the specific capacitance of the TiO_2_ was enhanced by ~2.3 times [4]. 

Among transition metal compounds, metal sulfides have been identified as the alternative material for supercapacitor electrode [13,14]. As compared to their metal oxides counterparts, metal sulfides show better performance for practical application due to the smaller volume change and higher efficiency. In particular, Fe_1−x_S nanostructures are emerging materials for supercapacitors and lithium-ion batteries due to their abundance in nature, cost-effectiveness, mechanical stability, and relatively large capacity compared with other metal sulfides [15,16,17,18]. However, low conductivity, sluggish kinetics, possible dissolution of sulfur, and severe volume change during the cyclic use are the issues of using iron sulfides in supercapacitor. Therefore, effective strategies should be developed to overcome these limitations for practical applications. One effective way to enhance the conductivity and buffer the volume expansion during the charge-discharge process is fabricating composite with conductive carbon materials especially, carbon nanofibers [18,19]. Taking all these factors into consideration, in this contribution, we prepared Fe_1−x_S-TiO_2_ decorated carbon nanofibers (Fe_1−x_S/TiO_2_/CNFs) by the electrospinning process followed by direct carbonization under an inert atmosphere of argon. The combination of Fe_1−x_S-TiO_2_ and conductive carbon nanofibers should be greatly beneficial to the structural ability and overall performance of the composite electrode. Such a novel structure exhibited enhanced electrochemical performance as compared to pristine TiO_2_ nanofibers.

## 2. Experimental

### 2.1. Materials

Iron (III) acetylacetonate, ethanol, polyvinylpyrrolidone (PVP), acetic acid, titanium tetraisopropoxide (97%), ammonium sulfide solution (40–48 wt % in H_2_O), and polyvinylidene fluoride (PVDF) were purchased from Sigma-Aldrich. *N*-methyl-2-pyrrolidone (NMP) (99.5%) was purchased from Showa Chemicals Inc., Tokyo, Japan. Potassium hydroxide (99.5%) was obtained from Samchun Pure Chemical Co. Ltd., Seoul, South Korea. All chemicals were of analytic grade and used without any purification. 

### 2.2. Synthesis of Fe_1−x_S-TiO_2_/CNFs Composite

**Figure 1:**
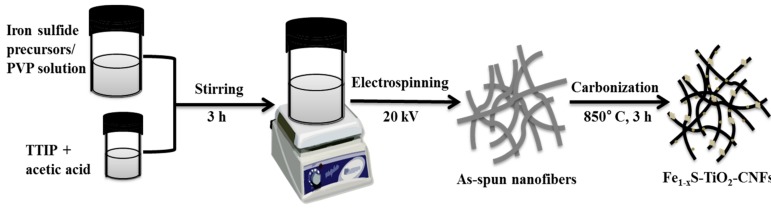
Schematic diagram showing the preparation of Fe_1−x_S-TiO_2_ nanoparticles embedded carbon nanofibers (Fe_1−x_S-TiO_2_/CNFs) composite.

Figure 1 depicts the synthetic protocol for the fabrication of Fe_1−x_S-TiO_2_/CNFs composite. All the chemicals in this experiment were purchased from Sigma-Aldrich. In the beginning, 0.5 g of PVP and 5 g of ethanol were taken in a glass vial and stirred for 2 h. Next, 0.45 g of Iron (III) acetylacetonate was mixed to the solution followed by the addition of 0.3 mL of ammonium sulfide and stirred for 1 h. In a separate vial, 1.5 g of titanium tetraisopropoxide was added to 3 g of acetic acid. After 10 min of stirring, it was transferred to the aforementioned PVP solution and stirred for 3 h at room temperature to form a homogeneous mixture. The electrospinning technique (NanoNC electrospinning system) was applied to fabricate nanofibers membrane from the resulting precursor/polymer solution. During the electrospinning process, the tip-to-collector distance and applied voltage were 15 cm and 18 kV, respectively. A polyethylene sheet was wrapped around the collector for collecting the nanofiber mat. The collected nanofibers mat was vacuum dried at 60 °C for 12 h. After carbonization in the argon atmosphere at 850 °C for 3 h, the sample was used for further analyses. For comparison, we prepared TiO_2_ NFs by following the same procedure as in our previous reports [4,20]. Briefly, the PVP solution containing titanium tetraisopropoxide was electrospun into the nanofiber under the identical conditions as mentioned above and the obtained nanofiber mat was subjected to calcination in air at 600 °C for 3 h to obtain TiO_2_ NFs.

### 2.3. Characterization

A morphological study of the sample was conducted by field emission scanning electron microscopy (FE-SEM, S-7400, Hitachi, Tokyo, Japan) and transmission electron microscopy (TEM, JEM-2010, JEOL, Tokyo, Japan). X-ray diffraction patterns (XRD) were collected at room temperature by using the X-ray diffractometer (Rigaku Co., Tokyo, Japan). The Fourier transform infrared (FTIR) spectra were recorded using an ABB Bomen MB100 spectrometer (Bomen, QC, Canada). The spectrum and origin were used to analyze and fit the FTIR spectra. The X-ray photoelectron spectroscopy (XPS) analyses were carried out by (XPS; Thermo Fisher Scientific, Loughborough, UK) and the data were fitted with origin. Brunauer–Emmett–Teller (BET) and Barrett–Joyner–Halenda (BJH) studies for the specific surface area and porosity of the sample were evaluated on an ASAP 2020 Plus system (Micromeritics Instrument Corp., Norcross, GA, USA).

### 2.4. Electrochemical Studies

The working electrodes were prepared by coating the nickel foam with a homogenous slurry of as-prepared samples, carbon black, and polyvinylidene fluoride (PVDF) at a weight ratio of 8:1:1 in *N*-methyl-2-pyrrolidone (NMP). The substrate was then dried at 60 °C overnight. The electrochemical performance was investigated with a conventional three-electrode electrochemical analyzer (VersaSTAT3, Algete-Madrid, Spain) at room temperature in a 2 M KOH solution as an electrolyte. Nickel foam, Ag/AgCl, and Pt wire were used as the working, reference, and counter electrodes, respectively. Cyclic voltammetry (CV), galvanostatic charge–discharge (GCD), electrochemical impedance spectroscopy (EIS), and the stability by charge–discharge cycles were performed using Versastate3. The CV tests were carried out at different scanning rates (10, 20, 30, 40, 50, 60, 70, 80, 90, and 100 mV/s) from 0.0 to 0.5 V. GCD tests were performed from 0 to 0.45 V at different current densities (1, 2, 3, 4, and 5 A/g). The EIS was measured from 100 kHz to 0.01 Hz. The specific capacitance of the electrodes was calculated by the GCD method by applying the equation below:

(1) $$C=\frac{I.\;\;∆t}{m.\;\;∆V}$$

where ‘*C’* is the specific capacitance (F/g), ‘*I’* is the charge-discharge current (A), ‘t’ is the discharge time (s), ‘m’ is the mass of the active material (g), and ‘V’ is the potential window (V).

## 3. Results and Discussion

**Figure 2:**
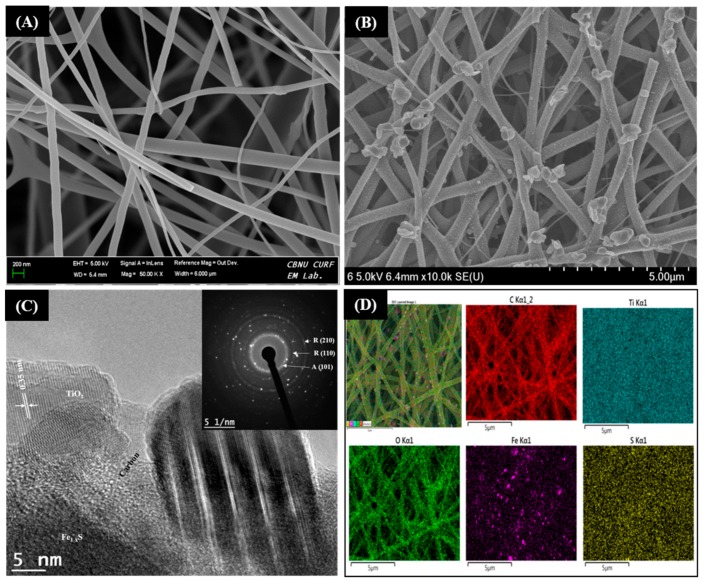
FE-SEM images of pristine TiO_2_ NFs (**A**) and Fe_1−x_S-TiO_2_/CNFs composite (**B**). (**C)** and (**D)** are the TEM image and elemental mapping of the Fe_1−x_S-TiO_2_-CNFs composite, respectively. The inset of (**C**) represents the SAED pattern.

Figure 2A,B depicts the FE-SEM images of TiO_2_ NFs and Fe_1−x_S-TiO_2_-CNFs composite, respectively. The pristine TiO_2_ NFs showed continuous and smooth morphology. It should be noted that the TiO_2_ nanofibers were prepared from titanium tetraisopropoxide/PVP nanofibers by calcination at 600 °C for 3 h. During the calcination process, the PVP was selectively removed and continuous TiO_2_ NFs were obtained. When the electrospun PVP nanofibers containing titanium tetraisopropoxide and iron sulfide precursors were directly carbonized under the argon atmosphere, the PVP was carbonized and titanium tetraisopropoxide was decomposed to give TiO_2_ NPs, thereby resulting in the formation of Fe_1−x_S-TiO_2_ incorporated carbon nanofibers (Figure 2B). Since the addition of sulfide ions in the iron precursor may result in the formation of iron chalcogenide, the nanofibers should have Fe_1−x_S-TiO_2_ particles embedded in the carbon matrix. It is noticeable that the nanofiber morphology was preserved even after the carbonization process. The TEM image in Figure 2C clearly shows the projection of particles from the body of carbon nanofiber. To confirm the successful loading of the iron sulfide-TiO_2_ NPs in the carbon nanofibers, elemental mapping was carried out. As in Figure 2D, the mapping study detected C, Ti, O, Fe, and S, showing the presence of all elements present in the fiber synthesis.

**Figure 3:**
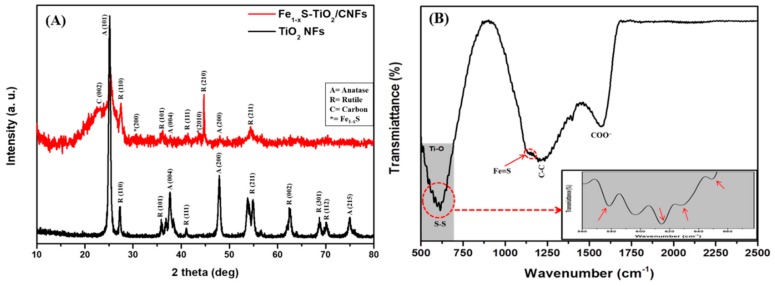
XRD spectra of Fe_1−x_S-TiO_2_/CNFs composite as compared to TiO_2_ NFs (**A**) and FTIR spectra of the Fe_1−x_S-TiO_2_/CNFs composite (**B**).

The phase structure and crystallinity of the as-obtained nanofibers were studied by XRD patterns, as shown in Figure 3A. The pristine TiO_2_ nanofibers showed both anatase and rutile phase. The apparent peaks for anatase were observed at 2θ values of 25.1, 38, 47.89, and 75° which were corresponding to the (101), (004), (200), and (125) phase, respectively [20]. Similarly, the existence of crystalline peaks at 2θ values of 27.3, 36, 41.2, 54.25, 62.4, 68.73, and 70.1° corresponding with the crystal plane of (110), (101), (111), (211), (002), (301), and (112), respectively represent the rutile phase of TiO_2_ NFs [20]. It should be noted that the anatase peaks are highly suppressed and the rutile peaks are dominant in the Fe_1−x_S-TiO_2_/CNFs composite sample. Moreover, a strong peak appeared at 2θ values of 44.7° which corresponds to (210) crystal plane of rutile TiO_2_. Previous studies have also shown that the crystallinity of TiO_2_ is affected by the temperature [4,21,22]. Since the synthesis of Fe_1−x_S-TiO_2_/CNFs was carried out at higher temperature (850 °C) as compared to TiO_2_ NFs (600 °C), the higher thermal treatment played a role in the transformation of the crystal phase from anatase to rutile [23]. In addition to the rutile peaks, a small and broad peak between 2θ values of 22 and 23° was observed, which is attributed to the (002) plane of the amorphous carbon [23,24]. This finding indicated that the PVP was successfully converted into the carbon under inert atmosphere during the carbonization process. Additionally, the peaks at 2θ values of 30.3 and 43.6° corresponding to the (200) and (2010) planes of Fe_1−x_S were observed (JCPDS # 29-0724) [25,26]. The selected area electron diffraction (SAED) in the inset in Figure 2C also shows the polycrystalline nature of the prepared sample. The interaction between the Fe_1−x_S, TiO_2_, and carbon was studied by Fourier transform infrared spectroscopy (FTIR) and the results are given in Figure 3B. The peaks at about 1200 and 1550 cm^−1^ are attributed to the C–C stretching vibration and asymmetric and symmetric stretching band of COO–, respectively [1,4]. The absorption peaks around 500–700 cm^−1^ for Ti–O vibration represents the interaction of TiO_2_ with the carbon [4]. The band at 1030 and 812 cm^−1^ are due to the asymmetric stretching of sulfur functional groups such as S–O [27]. Further, the peaks at 654, 623, and 582 cm^−1^ correspond to the disulfide (S–S) stretching vibrations [27]. It has also been reported in previous studies that the peaks around 1120–1156 cm^−1^ represent the pyrite surface chemistry (Fe=S) and the peaks around 607–622 cm^−1^ represent the stretching S-S and Fe-S bonds [28,29].

**Figure 4:**
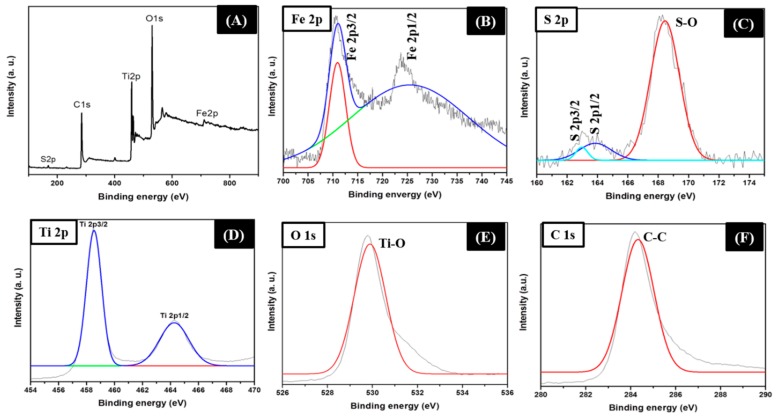
XPS survey (**A**) and high resolution XPS scan for Fe 2p (**B**), S 2p (**C**), Ti 2p (**D**), O 1s (**E**), and C 1s (**F**) of the Fe_1−x_S-TiO_2_/CNFs composite.

The surface composition and chemical state of the samples were further investigated by the X-ray photoelectron spectra (XPS). The survey spectrum indicated signals from Fe, S, Ti, O, and C in the composite sample (Figure 4A). In the high resolution of Fe 2p (Figure 4B), the peaks at ~710.9 and 724.3 eV are attributed to the Fe 2p3/2 and Fe 2p1/2, which are in good agreement with the Fe_1−x_S phase [25]. The peak at 710.9 eV suggests the existence of Fe^2+^ [17]. The high-resolution S 2p (Figure 4C) detected three peaks at ~163.4, 164.1, and 168. 7eV representing the characteristic feature of Fe_1−x_S [25]. The peaks at ~163.4 and 164.1 eV correspond to S 2p3/2 and S 2p1/2, respectively whereas the peak at 168.7 eV corresponds to the S-O bond [17,30]. The high resolution XPS scan of Ti 2p (Figure 4D) showed two peaks at about 458.5 and 464.2 eV, representing the Ti 2p3/2 and Ti 2p1/2, respectively [31]. The peak of O 1s (Figure 4E) is centered at ~529.9 eV, which is attributed to the oxide of the titanium [31]. In the XPS spectrum of C1s (Figure 4F), the peak centered at about 284.21 eV is associated with the C–C [32]. Overall, the obtained results indicated that Fe_1−x_S and TiO_2_ have been introduced into the carbon fiber matrix successfully.

**Figure 5:**
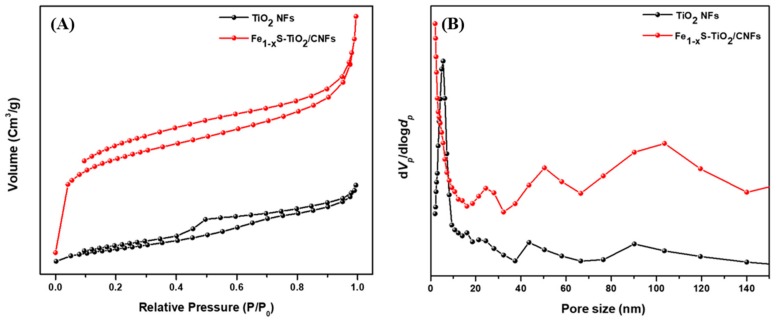
Nitrogen adsorption and desorption isotherm (**A**) and pore size distribution graph (**B**) for the Fe_1−x_S-TiO_2_/CNFs composite as compared to TiO_2_ NFs.

Materials with sufficient porosity and high surface area are considered to enhance electrochemical performance [33,34]. In order to study the specific surface area and the porosity of the synthesized materials, we performed Brunauer–Emmett–Teller (BET) gas sorption analyses (Figure 5A,B). The Nitrogen adsorption–desorption isotherm showed a mesoporous nature of the materials. It was noticed that the BET surface area and total pore volume of Fe_1−x_S-TiO_2_/CNFs composite were higher as compared to TiO_2_ NFs whereas the mean pore diameter was found to be lower (Table 1). The interconnected porous structure providing a larger specific surface area in Fe_1−x_S-TiO_2_/CNFs composite shortens the transport path and establishes a continuous pathway for the electrolyte diffusion [4]. Therefore, we expect a good electrochemical performance from the Fe_1−x_S-TiO_2_/CNFs composite sample.

**Figure 6:**
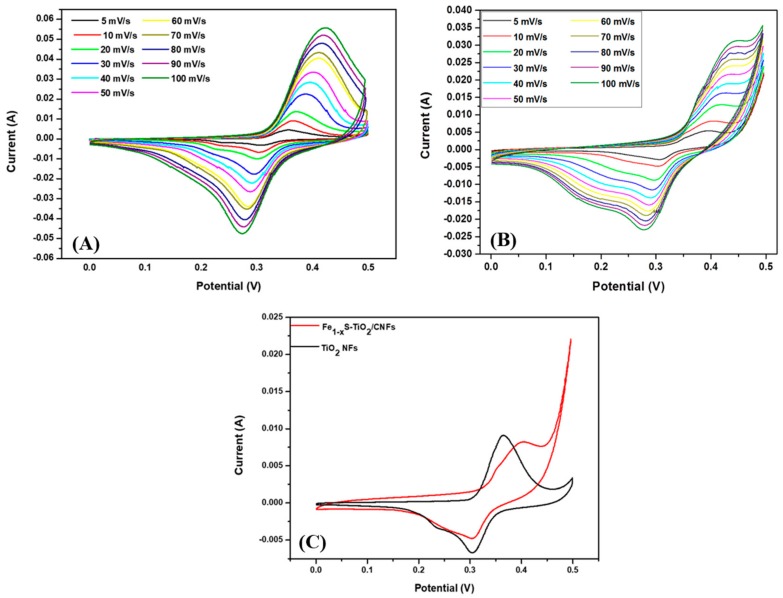
Cyclic voltammetry (CV) curves of TiO_2_ NFs (**A**) and Fe_1−x_S-TiO_2_/CNFs composite electrode (**B**) at various scan rates. (**C**) shows the CV results for the TiO_2_ NFs and Fe_1−x_S-TiO_2_/CNFs composite electrodes at a 10 mV/s scan rate.

**Figure 7:**
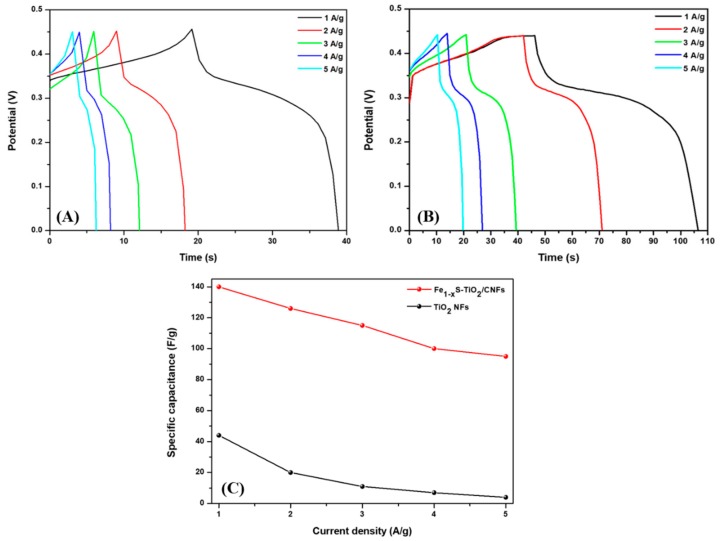
Galvanostatic charge–discharge (GCD) curves of TiO_2_ NFs (**A**) and Fe_1−x_S-TiO_2_/CNFs composite (**B**). (**C**) represents the specific capacitance of Fe_1−x_S-TiO_2_/CNFs composite electrode as compared to TiO_2_ NFs at various current densities.

**Figure 8:**
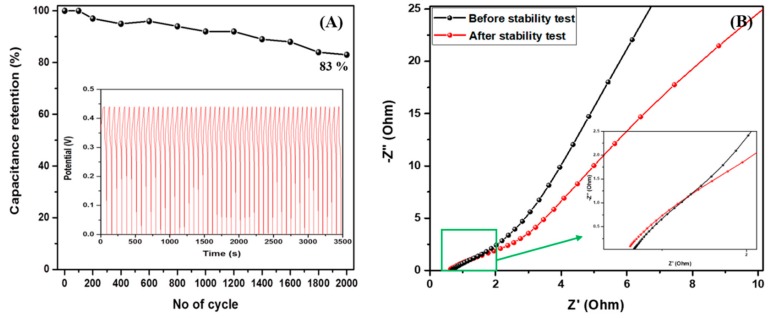
Cyclic stability for 2000 cycles (**A**) and EIS profile of the Fe_1−x_S-TiO_2_/CNFs composite electrode (**B**). Inset A represents GCD profiles for 50 cycles.

The cyclic voltammetry curves of the pristine TiO_2_ NFs and the composite sample were recorded in a 2 M KOH electrolyte solution in the voltage range of 0 to 0.5 V at various scan rates as shown in Figure 6A,B. The CV profile exhibited a pseudocapacitive nature in both cases; however, there is slight difference in the redox peaks which is due to the structural difference in the two electrodes. Figure 6C shows the comparative CV curves of the pristine TiO_2_ NFs and Fe_1−x_S-TiO_2_/CNFs at a scan rate of 10 mV/s. As in the figure, the area under CV curve of Fe_1−x_S-TiO_2_/CNFs is higher than that of pristine TiO_2_ NFs which suggests the higher capacitance of Fe_1−x_S-TiO_2_/CNFs as compared to TiO_2_ NFs. The addition of Fe_1−x_S and carbon in the TiO_2_ could have the combined effect of the faradiac capacitance of the Fe_1−x_S and double-layer capacitance of the carbon, thereby enhancing the electrochemical performance of the composite nanofiber. The specific capacitance of the as-fabricated composite materials was calculated by the GCD method and compared with pristine TiO_2_ NFs. The GCD was recorded over the potential window between 0 to 0.45 at the current density of 1, 2, 3, 4, and 5 A/g. It can be noted that the charge-discharge curves are consistent with the CV curves, suggesting a good pseudocapacitive behavior. The increase in the current density led to a decrease in discharge time (Figure 7A,B). The specific capacitances of TiO_2_ NFs at the current densities of 1, 2, 3, 4, and 5 were recorded as 44.2, 20.8, 11.3, 7.1, 4.3 F/g, respectively. The specific capacitances for the Fe_1−x_S-TiO_2_/CNFs composite were found to be 138.8, 126.3, 115.2, 100.4, and 95.4 F/g at the current densities of 1, 2, 3, 4, and 5 A/g, respectively (Figure 7C). The specific capacitance is mainly attributed to the porosity and conductivity, which are advantageous properties for the adsorption, penetration, and fast transportation of ions [33]. The cyclic stability is one of the important features of the electrode material in supercapacitor [1]. Therefore, we studied the stability of the Fe_1−x_S-TiO_2_/CNFs composite electrode by the GCD method for 2000 cycles and the results are given in Figure 8A. As in the figure, it can be observed that the capacitance retention is constant up to 100 cycles, whereas after 100 cycles, it is slightly decreased. The decline in capacitance retention pronounced more after 1000 cycles. The electrode material showed about 83% capacitive retention after 2000 cycles, suggesting good stability. The GCD curves showing the stability up to 50 cycles can be seen in the inset of Figure 8A. Next, the EIS were recorded (frequency range: 0.001–10,000 Hz) before and after the stability test to study the electrical conductivity of the electrode material (Figure 8B). As in the figure, after the stability test, the diameter of the semicircle at the high-frequency region is slightly increased as compared to that before the stability test, which suggests a slight reduction in the conductivity of the material [35,36]. Overall, it can be concluded that the as-synthesized Fe_1−x_S-TiO_2_/CNFs composite showed a good electrochemical performance showing some potentiality for the supercapacitor electrode. In order to compare the performance of the Fe_1−x_S-TiO_2_/CNFs electrode with some previously investigated TiO_2_-based electrode, a table is given (Table 2). It can be seen that the performance of the Fe_1−x_S-TiO_2_/CNFs as electrode material for supercapacitor is satisfactory.

## 4. Conclusions

In summary, the Fe_1−x_S-TiO_2_/CNFs composite was prepared by electrospinning followed by the carbonization under the inert atmosphere. The Fe_1−x_S-TiO_2_ loaded carbon nanofibers possessed high surface area, enough porosity, and good conductivity which provide a fast ion diffusion, thereby boosting electrochemical performances. This study suggested that the integration of Fe_1−x_S and TiO_2_ in the carbon fibers could be a good strategy to enhance the electrochemical behavior of the TiO_2_-based material.

## Figures and Tables

**Table 1:** Surface area, pore volume, and pore diameter of the Fe_1−x_S-TiO_2_/CNFs composite as compared to the TiO_2_ NFs.

Sample	BET Surface Area (m^2^/g)	Total Pore Volume (cm^3^/g)	Mean Pore Diameter (nm)
TiO_2_ NFs	44.507	0.07209	6.479
Fe_1−x_S-TiO_2_-CNFs	291.06	0.2402	3.3007

**Table 2:** Comparison of the electrochemical performance of as-synthesized Fe_1−x_S-TiO_2_/CNFs composite as a supercapacitor electrode material with some other TiO_2_-based electrodes.

S.N.	Electrode Material	Fabrication Method	Electrolyte	Specific Capacitance	Stability	Ref.
1	TiO_2_-nanotube-array	Electrochemical anodizaion	LiPF_6_ 1.2 M	5.12 mF/cm^2^ at 100 µA/cm^2^	88%	[37]
2	TiO_2_ NFs	Electrospinning	Li_2_SO_4_ 1 mol L^−1^	75 F/g at 1 mAg^−1^	95% after 5000 cycles	[38]
3.	KOH-treated TiO_2_ NFs	Electrospinning	Na_2_SO_4_ 1 mol L^−1^	65.84 F/g at 1 mV/s	78% after 10,000 cycles	[39]
4	TiO_2_@CNF	Electrospinning	KOH 6M	151.5 F/g at 1 A/g	97.8% after 4000 cycles	[40]
5	TiO_2_-CNFs	Electrospinning	KOH 2 M	106.57 F/g at 1 A/g	84% after 2000 cycles	[4]
6	Fe-TiO_2_/CNFs	Electrospinning	KOH 1 M	137 F/g at 5 mV/s	-	[41]
7	Fe_1−x_S-TiO_2_/CNFs	Electrospinning	KOH 2 M	138 F/g at 1 A/g	83% after 2000 cycles	This study

## References

[1] Pant B., Park M., Ojha G.P., Park J., Kuk Y.-S., Lee E.-J., Kim H.-Y., Park S.-J. (2018). Carbon nanofibers wrapped with zinc oxide nano-flakes as promising electrode material for supercapacitors. J. Colloid Interface Sci..

[2] Lee D.G., Kim B.-H. (2016). MnO2 decorated on electrospun carbon nanofiber/graphene composites as supercapacitor electrode materials. Synth. Metals.

[3] Lin S.-C., Lu Y.-T., Chien Y.-A., Wang J.-A., Chen P.-Y., Ma C.-C.M., Hu C.-C. (2018). Asymmetric supercapacitors based on electrospun carbon nanofiber/sodium-pre-intercalated manganese oxide electrodes with high power and energy densities. J. Power Sources.

[4] Pant B., Park M., Park S.-J. (2019). TiO2 NPs Assembled into a Carbon Nanofiber Composite Electrode by a One-Step Electrospinning Process for Supercapacitor Applications. Polymers.

[5] Afif A., Rahman S.M.H., Tasfiah Azad A., Zaini J., Islan M.A., Azad A.K. (2019). Advanced materials and technologies for hybrid supercapacitors for energy storage – A review. J. Energy Storage.

[6] Ojha G.P., Pant B., Park S.-J., Park M., Kim H.-Y. (2017). Synthesis and characterization of reduced graphene oxide decorated with CeO2-doped MnO2 nanorods for supercapacitor applications. J. Colloid Interface Sci..

[7] Ramadoss A., Kim G.-S., Kim S.J. (2013). Fabrication of reduced graphene oxide/TiO2 nanorod/reduced graphene oxide hybrid nanostructures as electrode materials for supercapacitor applications. CrystEngComm.

[8] Heng I., Lai C.W., Juan J.C., Numan A., Iqbal J., Teo E.Y.L. (2019). Low-temperature synthesis of TIO2 nanocrystals for high performance electrochemical supercapacitors. Ceramics Int..

[9] Yang S., Li Y., Sun J., Cao B. (2019). Laser induced oxygen-deficient TiO2/graphene hybrid for high-performance supercapacitor. J. Power Sources.

[10] Hsieh C.-T., Chang C.-C., Chen W.-Y., Hung W.-M. (2009). Electrochemical capacitance from carbon nanotubes decorated with titanium dioxide nanoparticles in acid electrolyte. J. Phys. Chem. Solids.

[11] Verma S., Sinha-Ray S., Sinha-Ray S. (2020). Electrospun CNF Supported Ceramics as Electrochemical Catalysts for Water Splitting and Fuel Cell: A Review. Polymers.

[12] Liu Y., Zeng Z., Bloom B., Waldeck D.H., Wei J. (2018). Stable Low-Current Electrodeposition of α-MnO2 on Superaligned Electrospun Carbon Nanofibers for High-Performance Energy Storage. Small.

[13] Ikkurthi K.D., Srinivasa Rao S., Jagadeesh M., Reddy A.E., Anitha T., Kim H.-J. (2018). Synthesis of nanostructured metal sulfides via a hydrothermal method and their use as an electrode material for supercapacitors. New J. Chem..

[14] Rui X., Tan H., Yan Q. (2014). Nanostructured metal sulfides for energy storage. Nanoscale.

[15] Chen Y., Xu S., Li Y., Jacob R.J., Kuang Y., Liu B., Wang Y., Pastel G., Salamanca-Riba L.G., Zachariah M.R. (2017). FeS2 Nanoparticles Embedded in Reduced Graphene Oxide toward Robust, High-Performance Electrocatalysts. Adv. Energy Mater..

[16] Karade S.S., Dwivedi P., Majumder S., Pandit B., Sankapal B.R. (2017). First report on a FeS-based 2 V operating flexible solid-state symmetric supercapacitor device. Sustainable Energy Fuels.

[17] Liu X., Yang Q., Mi M., Kong W., Ge Y., Ma J., Hu J. (2019). Fe1-xS/reduced graphene oxide composite as anode material for aqueous rechargeable Ni/Fe batteries. J. Alloys Comp..

[18] Liu Y., Fang Y., Zhao Z., Yuan C., Lou X.W. (2019). A Ternary Fe1−xS@Porous Carbon Nanowires/Reduced Graphene Oxide Hybrid Film Electrode with Superior Volumetric and Gravimetric Capacities for Flexible Sodium Ion Batteries. Adv. Energy Mater..

[19] Zhu Y., Fan X., Suo L., Luo C., Gao T., Wang C. (2016). Electrospun FeS2@Carbon Fiber Electrode as a High Energy Density Cathode for Rechargeable Lithium Batteries. ACS Nano.

[20] Pant B., Pant H.R., Park M., Liu Y., Choi J.-W., Barakat N.A.M., Kim H.-Y. (2014). Electrospun CdS–TiO2 doped carbon nanofibers for visible-light-induced photocatalytic hydrolysis of ammonia borane. Cata. Commun..

[21] Wetchakun N., Incessungvorn B., Wetchakun K., Phanichphant S. (2012). Influence of calcination temperature on anatase to rutile phase transformation in TiO2 nanoparticles synthesized by the modified sol–gel method. Mater. Lett..

[22] Wang H., Huang X., Li W., Gao J., Xue H., Li R.K.Y., Mai Y.-W. (2018). TiO2 nanoparticle decorated carbon nanofibers for removal of organic dyes. Colloids Sur. A Physicochem. Eng. Aspects.

[23] Pant B., Barakat N.A.M., Pant H.R., Park M., Saud P.S., Kim J.-W., Kim H.-Y. (2014). Synthesis and photocatalytic activities of CdS/TiO2 nanoparticles supported on carbon nanofibers for high efficient adsorption and simultaneous decomposition of organic dyes. J. Colloid Interface Sci..

[24] Pant B., Pant H.R., Barakat N.A.M., Park M., Jeon K., Choi Y., Kim H.-Y. (2013). Carbon nanofibers decorated with binary semiconductor (TiO2/ZnO) nanocomposites for the effective removal of organic pollutants and the enhancement of antibacterial activities. Ceramics Int..

[25] Wang C., Lan M., Zhang Y., Bian H., Yuen M.-F., Ostrikov K., Jiang J., Zhang W., Li Y.Y., Lu J. (2016). Fe1−xS/C nanocomposites from sugarcane waste-derived microporous carbon for high-performance lithium ion batteries. Green Chem..

[26] Argueta-Figueroa L., Torres-Gómez N., García-Contreras R., Vilchis-Nestor A.R., Martínez-Alvarez O., Acosta-Torres L.S., Arenas-Arrocena M.C. (2018). Hydrothermal synthesis of pyrrhotite (Fex-1S) nanoplates and their antibacterial, cytotoxic activity study. Pro. Natural Sci. Mater. Int..

[27] Venkateshalu S., Goban Kumar P., Kollu P., Jeong S.K., Grace A.N. (2018). Solvothermal synthesis and electrochemical properties of phase pure pyrite FeS2 for supercapacitor applications. Electrochimica Acta.

[28] Khabbaz M., Entezari M.H. (2016). Simple and versatile one-step synthesis of FeS2 nanoparticles by ultrasonic irradiation. J. Colloid Interface Sci..

[29] Middya S., Layek A., Dey A., Ray P.P. (2014). Synthesis of Nanocrystalline FeS2 with Increased Band Gap for Solar Energy Harvesting. J. Mater. Sci. Tech..

[30] Wang M.-H., Xue H.-G., Guo S.-P. (2019). In situ hydrothermal synthesis of rGO-wrapped Fe1−xS particles for lithium storage. J. Mater. Res..

[31] Wang X., Xiang Q., Liu B., Wang L., Luo T., Chen D., Shen G. (2013). TiO2 modified FeS Nanostructures with Enhanced Electrochemical Performance for Lithium-Ion Batteries. Sci. Reports.

[32] Liu Y., Zhong W., Yang C., Pan Q., Li Y., Wang G., Zheng F., Xiong X., Liu M., Zhang Q. (2018). Direct synthesis of FeS/N-doped carbon composite for high-performance sodium-ion batteries. J. Mater. Chem. A.

[33] Daubert J.S., Lewis N.P., Gotsch H.N., Mundy J.Z., Monroe D.N., Dickey E.C., Losego M.D., Parsons G.N. (2015). Effect of Meso- and Micro-Porosity in Carbon Electrodes on Atomic Layer Deposition of Pseudocapacitive V2O5 for High Performance Supercapacitors. Chem. Mater..

[34] Zhi M., Liu S., Hong Z., Wu N. (2014). Electrospun activated carbon nanofibers for supercapacitor electrodes. RSC Advances.

[35] Pant B., Park M., Park S.-J. (2019). MoS2/CdS/TiO2 ternary composite incorporated into carbon nanofibers for the removal of organic pollutants from water. Inorg. Chem. Commun..

[36] Pant B., Ojha G.P., Kim H.-Y., Park M., Park S.-J. (2019). Fly-ash-incorporated electrospun zinc oxide nanofibers: Potential material for environmental remediation. Environmental Pollution.

[37] Ahmed F., Pervez S.A., Aljaafari A., Alshoaibi A., Abuhimd H., Oh J., Koo B.H. (2019). Fabrication of TiO2-Nanotube-Array-Based Supercapacitors. Micromachines.

[38] Kuchi C., Narayana A.L., Hussain O.M., Reddy P.S. (2020). Electrospun TiO2 nanofiber electrodes for high performance supercapacitors. Mater. Res. Express.

[39] He X., Yang C.P., Zhang G.L., Shi D.W., Huang Q.A., Xiao H.B., Liu Y., Xiong R. (2016). Supercapacitor of TiO2 nanofibers by electrospinning and KOH treatment. Mater. Des..

[40] Tang K., Li Y., Cao H., Su C., Zhang Z., Zhang Y. (2016). Amorphous-crystalline TiO2/carbon nanofibers composite electrode by one-step electrospinning for symmetric supercapacitor. Electrochimica Acta.

[41] Tolba G., Motlak M., Bastaweesy A.M., Ashour E.A., Abdelmoez W., El-Newehy M., Barakat N. (2015). Synthesis of Novel Fe-doped Amorphous TiO_2_/C Nanofibers for Supercapacitors Applications. Int. J. Electrochem. Sci..

